# Isolation and pathogenicity of a highly virulent group III porcine Getah virus in China

**DOI:** 10.3389/fcimb.2024.1494654

**Published:** 2024-10-17

**Authors:** Yu Wu, Xiaopeng Gao, Zhanpeng Kuang, Limiao Lin, Hao Zhang, Lijuan Yin, Jiabing Hong, Bohua Ren, Qunhui Li, Lianxiang Wang

**Affiliations:** ^1^ Wen’s Group Academy, Wen’s Foodstuffs Group Co., Ltd., Xinxing, Guangdong, China; ^2^ State Key Laboratory of Biocontrol, Guangzhou Higher Education Mega Center, School of Life Sciences, Sun Yat-sen University, Guangzhou, China; ^3^ College of Animal Science, South China Agricultural University & Guangdong Provincial Key Lab of Agro-Animal Genomics and Molecular Breeding, Guangzhou, China

**Keywords:** Getah virus, isolation, pathogenicity, whole-genome sequencing, piglets and sows

## Abstract

**Introduction:**

Getah virus (GETV) is a multi-host virus found in pigs, horses, and blue foxes. Clinically, GETV can cause fever, diarrhea, and reproductive disorders in pigs, representing significant threats to pig breeding. At present, few studies have examined the pathogenicity of GETV in pigs of different ages.

**Methods:**

In the present study, a new strain, named GETV-QJ, was isolated from clinically ill pigs, and whole genome sequencing analysis was performed. Besides the pathogenicity of piglets and pregnant sows of this strain was further studied.

**Results:**

the results illustrated that the strain belonged to group III. The strain had 93.6%–96.3% homology with other subtypes, and its homology with the same subtype strain ranged 96.5%–99%. Further studies on the pathogenicity of the virus indicated that this strain caused severe diarrhea, fever, and intestinal and lung damage in 7-day-old piglets, resulting in their death. The piglet survival rate was 0%. In pregnant sows, this strain did not cause fever, death, or abortion, but it induced viremia, which affected the farrowing performance of sows and led to reduced piglet survival.

**Discussion:**

In this study, we isolated a highly virulent group III and comprehensively established a pathogenic model of GETV in piglets and sows, providing a reference and guidance for the prevention and control of this infection.

## 1 Introduction

Getah virus (GETV) is a mosquito-vectored, single-stranded, positive-strand RNA virus belonging to the genus Alphavirus in the family Togaviridae (Kono et al., 1980). Since GETV was first isolated in Malaysia in 1955, it has been widely detected across Eurasia and the Pacific, including China, Russia, Japan, and Australia (Fukunaga et al., 2000). GETV has a wide host range, and it can cause disease in pigs, horses, cattle, and blue foxes (Kawamura et al., 1987; Yago et al., 1987; Kuwata et al., 2018; Liu et al., 2019; Rattanatumhi et al., 2022). GETV can also infect several laboratory animal species, including rabbits, apes, orangutans, and guinea pigs. Meanwhile, serum samples from animals such as chickens, ducks, cattle, wild pigs, goats, and kangaroos contained neutralizing antibodies against GETV (Shi et al., 2019). Death occurred 3 days after intramuscular injection of the virus in 5-day-old piglets, but no clinical symptoms were observed in pigs that did not produce GETV antibodies at 4–5 months of age (Kawamura et al., 1987). This suggests that newborn animals are more susceptible to GETV infection, which could result in more severe symptoms.

The length of the GETV genome is approximately 11,690 nucleotides, and it contains two open-reading frames (ORFs). The first ORF contains 7407 nucleotides, and it primarily encodes a series of nonstructural proteins (Nspl–Nsp4) (Zhai et al., 2008). The second ORF contains 3759 nucleotides, and it encodes a variety of structural proteins (Cap, E3, E2, 6K, and E1) used to form viral particles, which work together in viral transcription, viral replication, and resistance mechanisms in host cells (Jose et al., 2009; Tan et al., 2022). A 26S rRNA ligation region located between the two ORFs facilitates the transcription of 26S rRNA in the intracellular subgenome. A 72-nucleotide methylated cap knot and a 411-nucleotide poly(A) tail are located at the 5′ and 3′ ends, respectively, of the genome. The capsid protein, as a structural protein, helps the virus replicate in cells (Kamada et al., 1982). E2 gene, as another key structural protein, constitutes the envelope of virions, and it adsorbs onto target cells, facilitates host infection, and induces anti-infection immunity. The in-depth study of capsid and E2 proteins is helpful for clarifying the biological characteristics and infection mechanism of GETV, and such data could provide new ideas and methods for the research and development of antiviral treatments and vaccines (Weger-Lucarelli et al., 2015).

In 1964, GETV was first isolated in Hainan Province, China, and the virus was subsequently detected in many provinces (Ren et al., 2020; Mohamed-Romai-Noor et al., 2022; Gao et al., 2023). After 2006, the distribution range of GETV expanded from 6 to 16 provinces, and the range of infected animals extended from horses, pigs, and other domestic animals to foxes, cattle, and other animals (Kamada et al., 1991; Xing et al., 2020). In addition, GETV-specific antibodies have been detected in human serum, indicating that the scope of GETV infection has expanded and the impact on public health cannot be ignored. GETV infection has been reported in piglets, causing diarrhea and death, whereas reproductive disorders have been observed in sows, with approximately 1500 piglets dying in four intensive farms in Guangxi, Hubei, Shandong, and Henan since May 2019, highlighting the serious potential impact of GETV infection in China (Yang et al., 2018; Lu et al., 2019).

In this study, a GETV strain was detected and isolated from the spleen and intestinal tissues of aborted fetuses in a pig farm in Shanxi Province, China, and whole-genome sequence determination and pathogenicity evaluation of the virus were performed to analyze its molecular genetic characteristics and pathogenicity.

## 2 Materials and methods

### 2.1 Sampling and virus isolation

The collected tissue samples were placed in a 2-mL centrifuge tube. One milliliter of Dulbecco’s modified Eagle’s medium (DMEM) was added, and the tube was triturated, subjected to repeated freeze–thaw cycles, and centrifuged at 12,000 × *g* for 10 min. The supernatant was transferred to a new 1.5-mL centrifuge tube and stored at −80°C.

The supernatant of positive samples was mixed with DMEM at a ratio of 1:5, filtered, and sterilized through a 0.22-μm filter. One milliliter of this mixture was aspirated and seeded on a monolayer of PK-15 cells in a six-well plate. The plate was incubated for 1 h, and the supernatant was discarded. Two milliliters of DMEM containing 2% fetal bovine serum and 1% bispecific antibody were added, and the plate was incubated in a 37°C, 5% CO_2_ incubator for 48 h to observe cytopathic effects. If no lesions were present, then the cells were subjected to three freeze–thaw cycles at −80°C. The cells were then collected and passaged until approximately 80% of the cells displayed lesions. During passaging, positive-control wells in which GETV was inoculated and negative-control wells without virus inoculation were created.

### 2.2 Reverse transcription-polymerase chain reaction amplification and GETV genome sequencing

Total RNA was extracted from cells using the FastPure Viral DNA/RNA Mini Kit. RT was performed using HiScript III 1st Strand cDNA Synthesis Kit reagent. All primers used to amplify GETV genomic fragments were designed and stored in our laboratory. RT and PCR were performed using the PrimeScript™ RT-PCR Kit (Promega, Madison, WI, USA) and PrimeSTAR^®^ GXL DNA Polymerase, respectively. The PCR products were cloned into the pMD19-T vector using the TOPO^®^ TA Cloning Kit (Invitrogen, Waltham, MA, USA) and submitted to Sangon Biotech (Shanghai, China) for sequencing.

### 2.3 Sequence alignments and phylogenetic analysis

To analyze the genome-wide phylogenetic relationships of GETV, we obtained usable sequences of GETV strains from the US National Center for Biotechnology Information (Bethesda, MD, USA). Matrix alignment was established using MEGA 7 software, and the aligned matrix data were analyzed. The Tamura–Nei model and the maximum likelihood method of gamma distribution were used to infer the evolutionary history. The percentage of replicates that were clustered together in the bootstrap test (1000 replicates) is presented next to the branches of each tree.

### 2.4 Electron microscopic analysis

GETV-QJ was seeded into PK-15 cells in a T75 flask, and when the lesion rate was 100%, the cell supernatant was collected. After ultracentrifugation, virions were collected for electron microscopy. Virus-containing supernatants were negatively stained and examined using a transmission electron microscope.

### 2.5 Histopathology

At autopsy, intestinal and lung tissues were collected, photographed, fixed with 4% paraformaldehyde, and stained with hematoxylin and eosin for immunohistochemical analysis. Staining was performed automatically by a Leica automatic dyeing machine.

### 2.6 Animal experiment design

To assess the pathogenicity of GETV-QJ, we performed challenge tests in piglets. Twelve 7-day-old healthy piglets were divided into two groups (six piglets each). Approximately 2 mL of GETV-QJ (1 × 10^6^ TCID_50_) was intramuscularly injected, and negative-control piglets were inoculated with DMEM. Rectal temperature, diarrhea (Lin et al., 2017), and other clinical signs were recorded daily in each group. Serum samples were collected weekly to measure viral loads and antibody levels. The survival of the piglets was recorded during the experiment. In the sow pathogenicity test, eight pregnant sows were randomly divided into two groups and challenged at 85 days of gestation. After challenge, the body temperature and viral load of pregnant sows were measured. Finally, the farrowing of the pregnant sows was observed and recorded.

### 2.7 Statistical analysis

In each experimental group, statistical significance was measured using one-way analysis of variance. Two-sided P < 0.05 indicated statistical significance.

### 2.8 Ethical approval

Our animal experiments were approved by the Animal Ethics Committee of South China Agricultural University and conducted under the guidance of the South China Agricultural University Institutional Animal Care and Use Committee (SCAU-AEC-2023C043).

## 3 Results

### 3.1 Virus isolation

**Figure 1:**
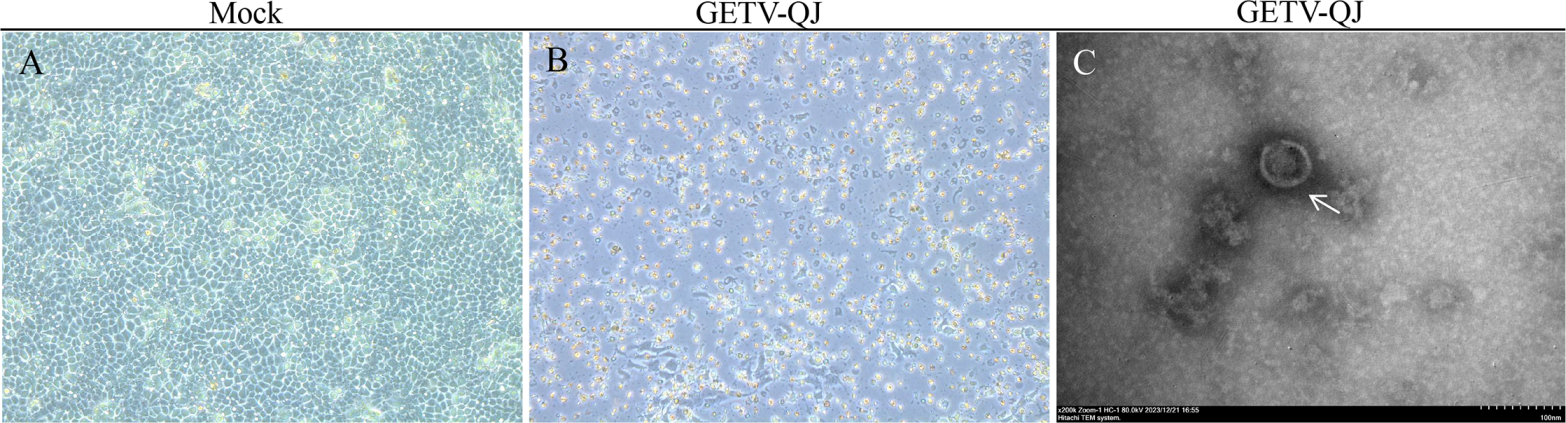
Strain isolation and identification. **(A, B)** Mock and virus inoculation in PK-15 cells. The slides were observed using a microscope at 200×. **(C)** Electron micrograph of SL-01 (indicated by arrows) Magnification, 200,000×.

Following the collection and detection of clinical samples, positive samples were screened. In this study, blood positive samples were used for virus isolation in PK-15 cells. Stable cytopathic effects were obtained in PK-15 cells via successive culture. Then, the virus solution was collected for ultraionization(**Figures 1A, B**). After treatment, electron microscopy revealed that the virions were approximately 70 nm in size. The isolated strain was named GETV-QJ (**Figure 1C**).

### 3.2 Genome-wide analysis of GETV-QJ

**Figure 2:**
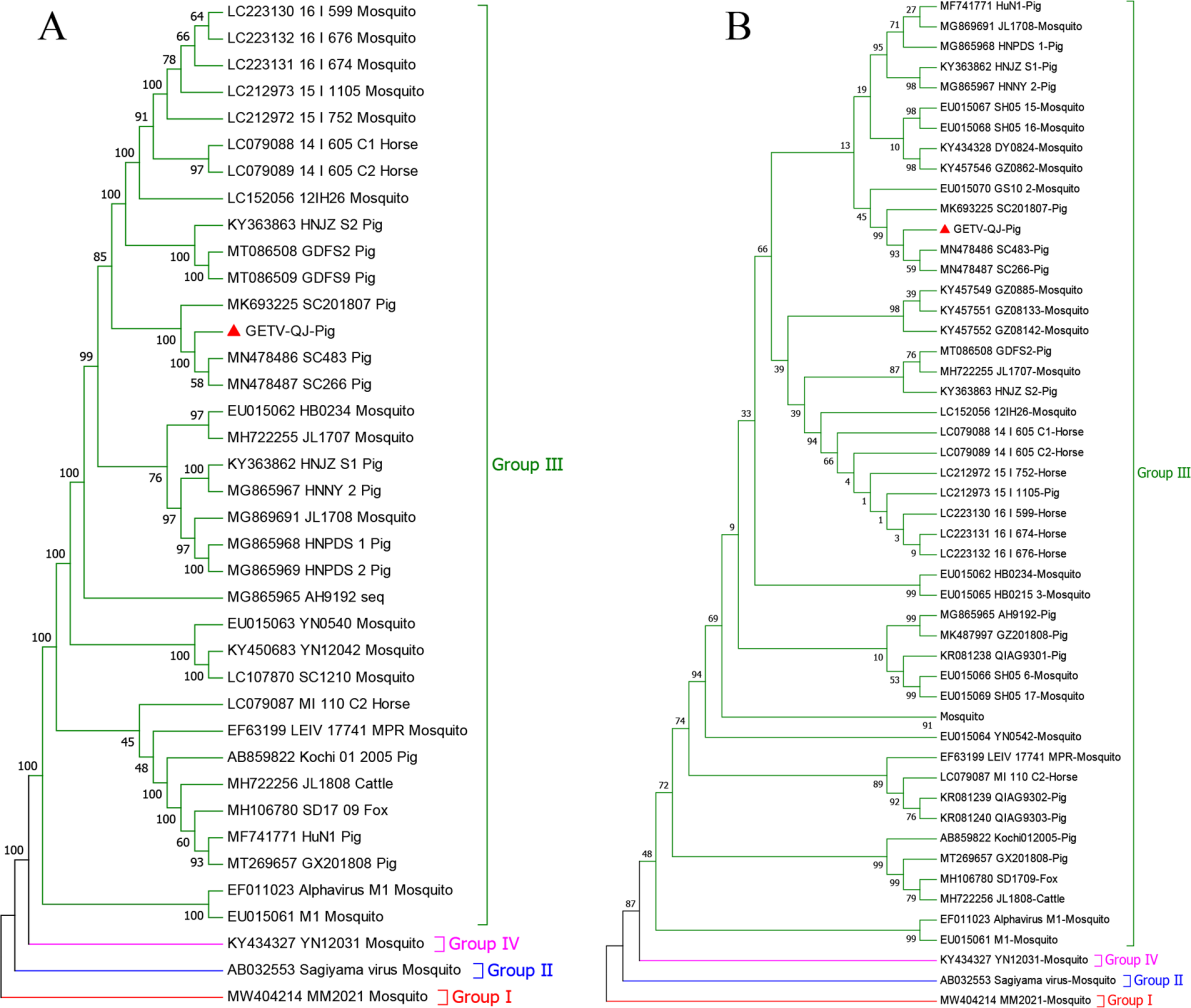
Phylogenetic analysis of GETV-QJ. **(A)** Phylogenetic trees constructed based on whole-length SL-01 genomes. **(B)** Phylogenetic trees constructed based on the E2 gene of GETV-QJ.

Whole-genome sequencing revealed that the genome of the GETV-QJ strain was 11689 bp in length (GenBank accession number: PQ289630). First, nucleotide similarity analysis was performed using the classic strains I, II, and IV as well as eight type III porcine strains. The results indicated that the strain had 93.6% homology with MM2021 mosquito type I, 96.3% homology with Sagiyama virus mosquito type II, and 95.4% homology with YN12031 mosquito type IV. The homology with the eight type III swine strains ranged 96.5%–99% (**Table 1**). At the same time, the GETV-QJ strain was classified as a type III strain based on the results of genetic and evolutionary analyses using the whole genome and E2 gene (**Figure 2**).

**Table 1:** Nucleotide and amino acid sequence and identity analyses of GETV-QJ and the other GETV strains.

Virus isolates	GETV-QJ		
	Complete genome	E2 Nucleotide	E2 Amino acid
MM2021-Mosquito	93.6	93.6	97.2
Sagiyama-virus-Mosquito	96.3	96.8	98.1
YN12031-Mosquito	95.4	96.2	98.1
GX201808-Pig	96.5	96.5	98.3
HuN1-Pig	96.7	99.0	99.8
GDFS2-Pig	98.0	99.1	99.5
SC266-Pig	99.0	99.8	99.5
AH9192-Pig	97.8	98.4	99.1
HNJZS1-Pig	98.2	98.8	99.8
Kochi012005-Pig	96.9	97.4	98.8
HNPDS1-Pig	98.2	99.0	99.8

### 3.3 GETV-QJ E2 homology and amino acid analysis

**Figure 3:**
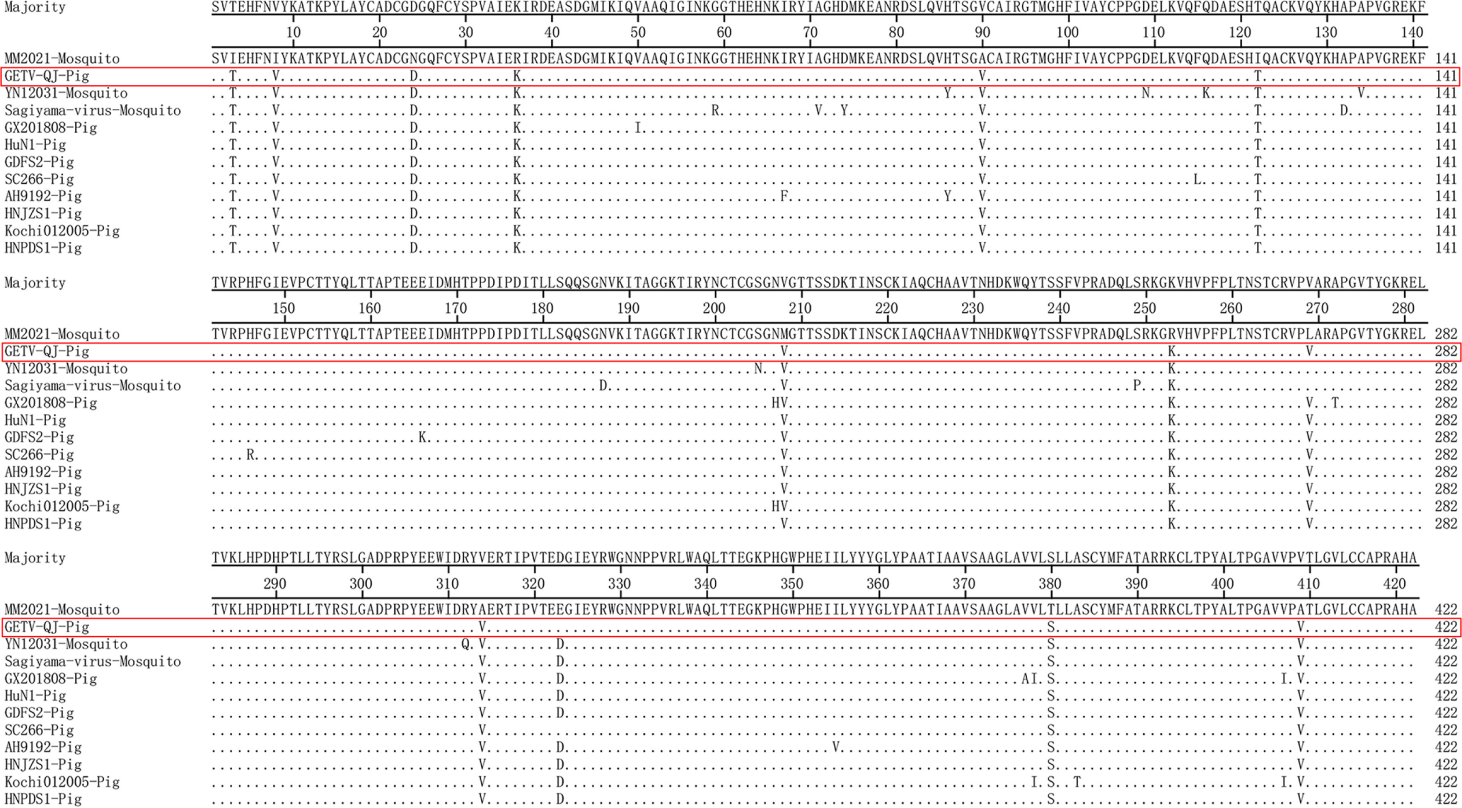
Amino acid sequence alignment based on E2.

The nucleotide and amino acid similarities of the GETV-QJ E2 gene with those of the I, II, and IV strains and eight type III swine strains were analyzed. The nucleotide homology between the GETV-QJ E2 gene and those of the representative strains ranged 93.6%–99.1%, and the amino acid homology ranged 97.2%–99.8% (**Table 1**). Further amino acid mutation analysis revealed that all GETV E2 genes were 1266 nucleotides in length, and they encoded 422 amino acids. Compared with the type I strain MM2021 mosquito, GETV-QJ isolates shared 12 common differential amino acid sites, which were located at E3 (I to T), E8 (I to V), E24 (N to D), E36 (R to K), E90 (A to V), E122 (I to T), E208 (M to V), E253 (R to K), E269 (L to V), E314 (A to V), E380 (T to S), and E409 (A to V) (**Figure 3**).

### 3.4 Pathogenicity analysis of piglets

**Figure 4:**
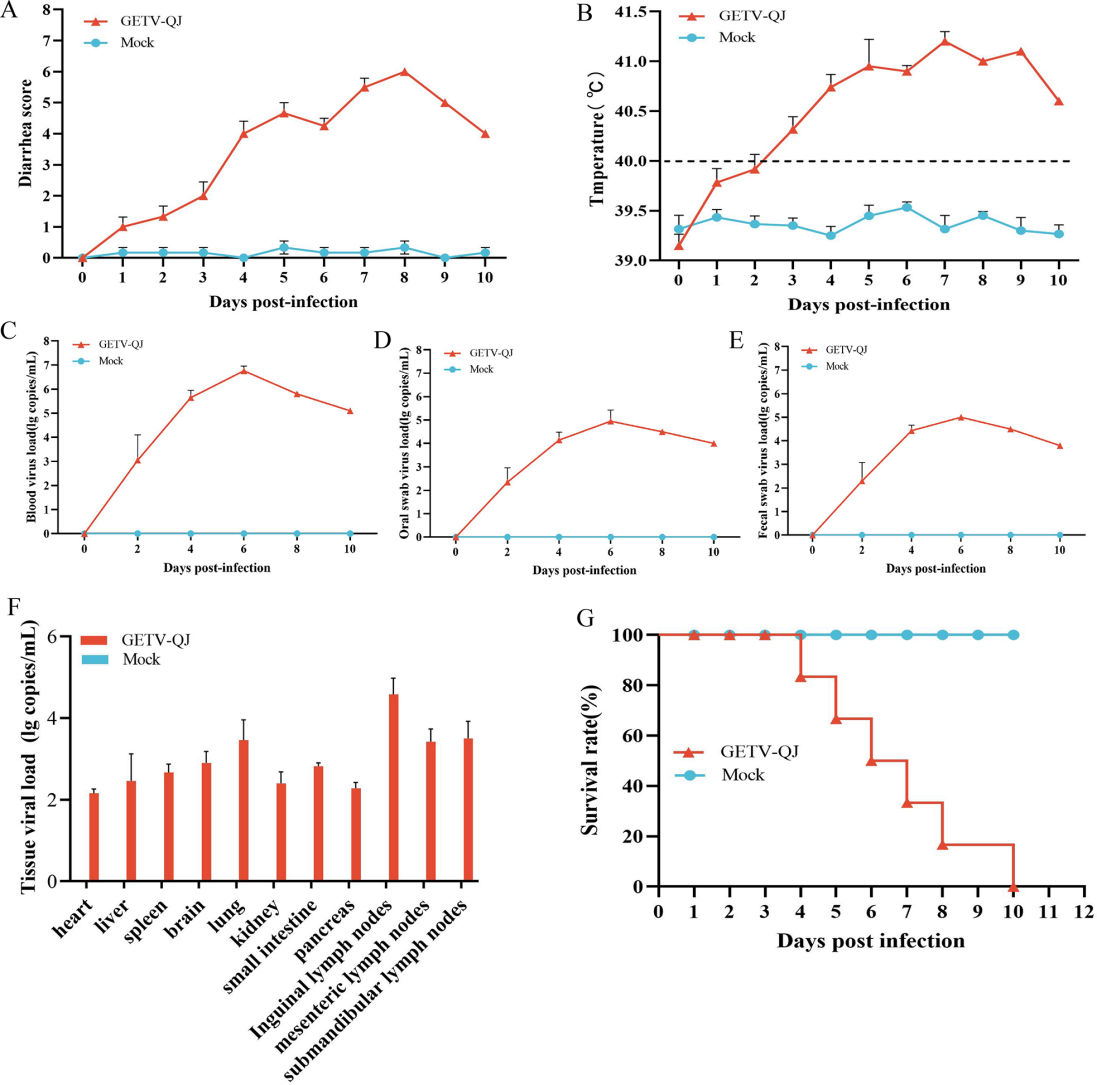
Pathogenicity results in piglets. **(A)** Diarrhea score of piglets after challenge throughout the experiment. **(B)** Body temperature changes in piglets in each group after challenge. **(C–E)** Viral load detection in the blood, oral swabs, and fecal swabs. **(F)** Different tissues viral loads. **(G)** Survival rate of pigs in each group during the challenge study. Each bar represents the mean ± standard deviation in each group.

**Figure 5:**
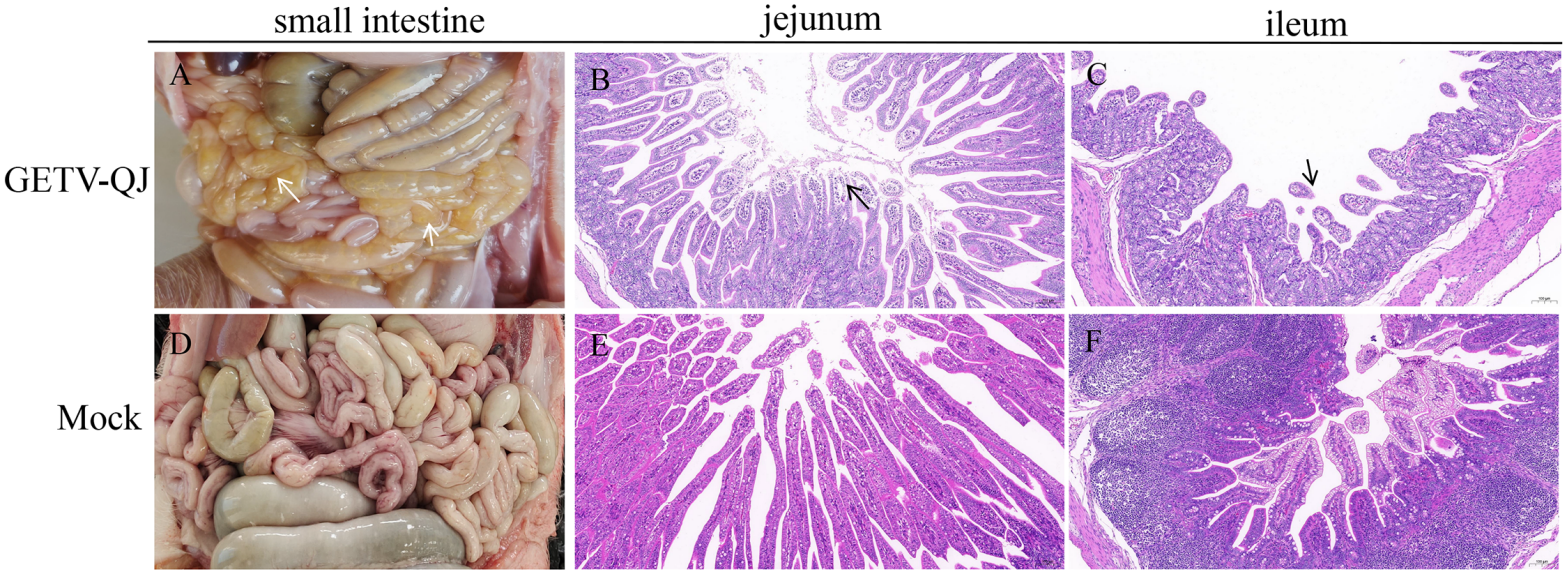
Observation and detection via pathological necropsy. **(A, B)** Comparison of small intestinal lesions in different groups at necropsy. Lesion sites were marked with white arrows. **(C–F)** H&E-stained jejunum tissue sections of the different groups. Lesion sites were marked with black arrows.

**Figure 6:**
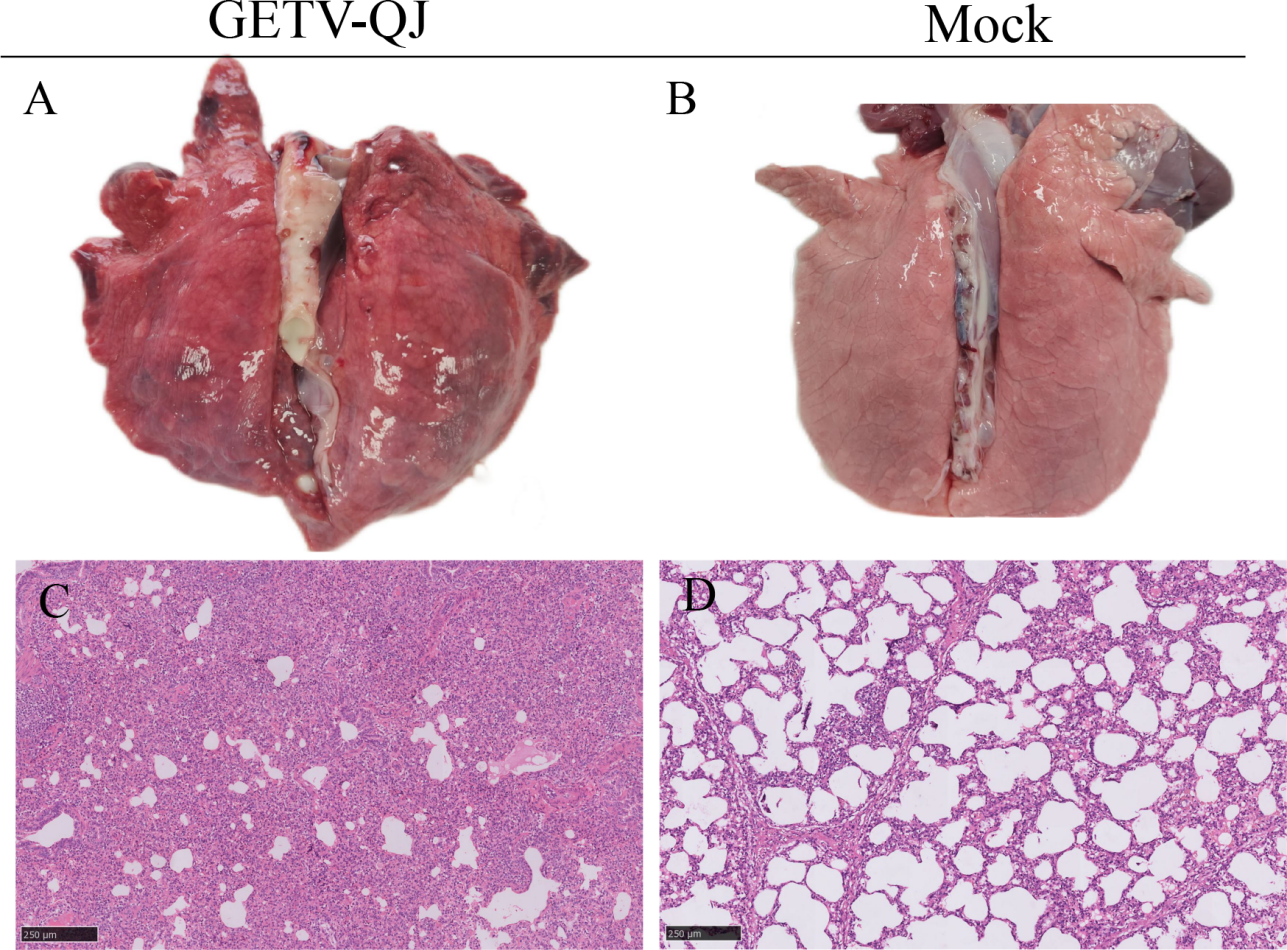
Observation and detection via pathological necropsy. **(A, B)** Lung necropsy observation: the lung tissue showed significant consolidation and diffuse hemorrhage after GETV-QJ challenged. **(C, D)** Histopathology tests: alveolar cell inflammatory infiltrate.

In pathogenicity analysis, the piglets had obvious diarrhea on the third day after viral challenge, and the diarrhea score was higher in infected piglets than in mock-inoculated piglets (**Figure 4A**). At the same time, the pigs had fever, and the average body temperature remained above 41.5°C on the fifth day after challenge (**Figure 4B**). Further detoxification tests revealed the presence of large amounts of the virus in mouth swabs, anal swabs, and whole blood, and the strain caused multiorgan infection in pigs with a high viral tissue load (**Figures 4C–F**). Meanwhile, tissue autopsy illustrated that the small intestine of infected piglets was obviously thinned and filled with yellow watery content (**Figures 5A, B**). In addition, there was significant bleeding and lesions in the lungs (**Figures 6A, B**). Further histopathological biopsy revealed shedding and atrophy of the epithelial villous cells of the jejunoileum (**Figures 5C–F**), and the piglets had inflammatory infiltration in their lungs (**Figures 6C, D**). More obviously, the pigs began to die on the fourth day after challenge, and all pigs in the challenge group died by day 10. Conversely, no pigs died in the mock group (**Figure 4G**).

### 3.5 Pathogenicity analysis of pregnant sows

**Figure 7:**
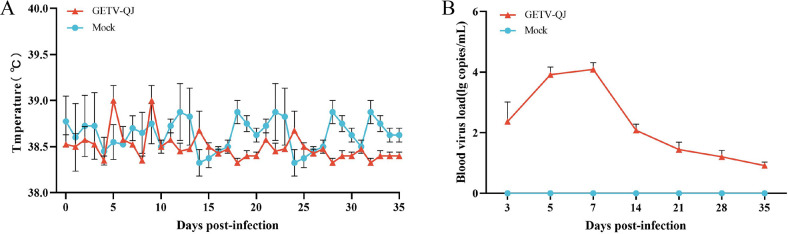
Pathogenicity results in pregnant sows. **(A)** Body temperature changes in pregnant sows in each group after challenge. **(B)** Viral load detection in the blood. Each bar represents the mean ± standard deviation in each group.

The pathogenicity of the GETV-QJ strain was further evaluated via challenge test in pregnant sows. After viral challenge, the pregnant sows did not exhibit clinical symptoms such as diarrhea and fever (**Figure 7A**). The detoxification test revealed a high viral load in the whole blood after viral challenge, and detoxification lasted for approximately 14 days (**Figure 7B**). Finally, the assessment of farrowing in pregnant sows indicated the absence of sow death and abortion after viral challenge, but the number of weak and stillborn babies was higher in the challenge group than in the mock group. The survival rate of piglets in the challenge group was 76.5%, versus 90.5% in the mock group (**Table 2**).

**Table 2:** Sows delivery data after challenge.

Groups	Mock	GETV-QJ
Challenge virus	DMEM	Virus cultures
Total born	15/11/13/14	15/12/9/11
Live born	14/11/11/12	11/9/7/9
Stillborn	0/0/0/0	4/3/2/2
Mummified	0/0/0/0	0/0/0/0
Light (< 1 kg)	1/0/2/2	2/3/3/1
Piglet survival	90.5%	76.5%
Sow amblosis	0/4(0%)	0/4(0%)
Sow survival	4/4(100%)	4/4(100%)

## 4 Discussion

As of 2018, GETV was detected in 18 provinces in different hosts in China. GETV has gradually evolved into four evolutionary groups (groups I–IV) (Yang et al., 2018; Sam et al., 2022). MM20021, the original strain isolated from Malaysia mosquitoes, is the only strain in group I. Group III includes a strain isolated in Japan more than 60 years ago (SAGV) (Shin et al., 2012). Group IV is a new evolutionary group that emerged 30 years ago, and its representative strain is YN12031. Most strains circulating globally belong to group III (Shi et al., 2022a, b). In addition, all GETV strains isolated from pigs to date belong to group III. The genome length of the GETV-QJ strain isolated in this study was 11689 bp, and the whole genome and E2 gene were classified into the Sanya lineage. The homology of GETV-QJ with other subtypes was 93.6%–96.3%, and that within the same subtype ranged 96.5%–99%, indicating that the genome-wide homology of porcine strains was generally high and the sequences of strains were relatively stable.

E2 is the main key protein of alphaviruses, and it plays an extremely important role in the life cycle of viruses by helping them attach to and enter host cells, eliciting host immune responses, and participating in virus replication, assembly, and budding (Zhang et al., 2011; Kim and Diamond, 2023; Zhao et al., 2023). In this study, the amino acid sequence of GETV E2 protein was differentially analyzed in comparison with that of the type I strain MM2021 mosquito. In total, 12 differential amino acid sites were found in the GETV-QJ strain at loci 3, 8, 24, 36, 90, 122, 208, 253, 269, 314, 380, and 409. Changes in these loci might be related to the continuous adaptation of GETV to new hosts and expansion of its spread. Previous studies revealed that substitution of amino acid residue 253 attenuates viral virulence, and this residue is part of the heparan sulfate binding site, which is ubiquitous on the cell surface, suggesting that this site plays a key role in cellular invasion by the virus. Further experimental verification is needed to clarify the roles of the other sites. Such research will provide a deeper understanding of the pathogenic mechanism of the virus and a theoretical basis for the development of specific drugs and vaccines.

Clinically, there are some differences in the symptoms of GETV infection in pigs of different ages. Newborn piglets infected with the virus have severe symptoms, including brownish–yellow diarrhea, anorexia, redness of the skin, tongue tremors, limb incoordination, and death (Kim and Diamond, 2023; Zhao et al., 2023; Li et al., 2017). Piglets older than 10 days of age might exhibit mental exhaustion, fever, loss of appetite, and a significant decrease in daily weight gain (Zhao et al., 2023). Some piglets have a sudden onset of illness, including dyspnea and neurological symptoms, leading to death after a short period. After sows are infected with GETV, they mainly display symptoms of reproductive disorders, including abortion and stillbirth. These symptoms can lead to a significant decrease in the reproductive performance of the sows, which can have a serious impact on the economic performance of the farm. Some sows might also exhibit moderate anorexia and a lack of appetite for 1–2 days (Ren et al., 2020), but mortality is usually not significant. At present, there are few studies on the pathogenicity of GETV isolated from pigs. Therefore, this study completely established the pathogenic model of the GETV-QJ isolate in piglets and pregnant sows. The results of viral challenge revealed that this strain can cause severe diarrhea, fever, multiorgan infection, and intestinal and lung damage in 7-day-old piglets, resulting in the mortality rate of 100%. In pregnant sows, this strain did not cause fever, death, or abortion, but it induced viremia and affected farrowing performance, resulting in reduced piglet survival.

## 5 Conclusions

In general, the type III strain GETV-QJ was isolated from pigs. This strain was highly pathogenic to piglets, causing severe diarrhea, fever, and death in all infected animals. Conversely, the strain was weakly pathogenic to sows, but it reduced their farrowing performance. This study built a complete pathogenic model of GETV infection in piglets and sows, thereby providing reference and guidance for its prevention and control.

## Data Availability

The datasets presented in this study can be found in online repositories. The names of the repository/repositories and accession number(s) can be found in the article/Supplementary Material.
